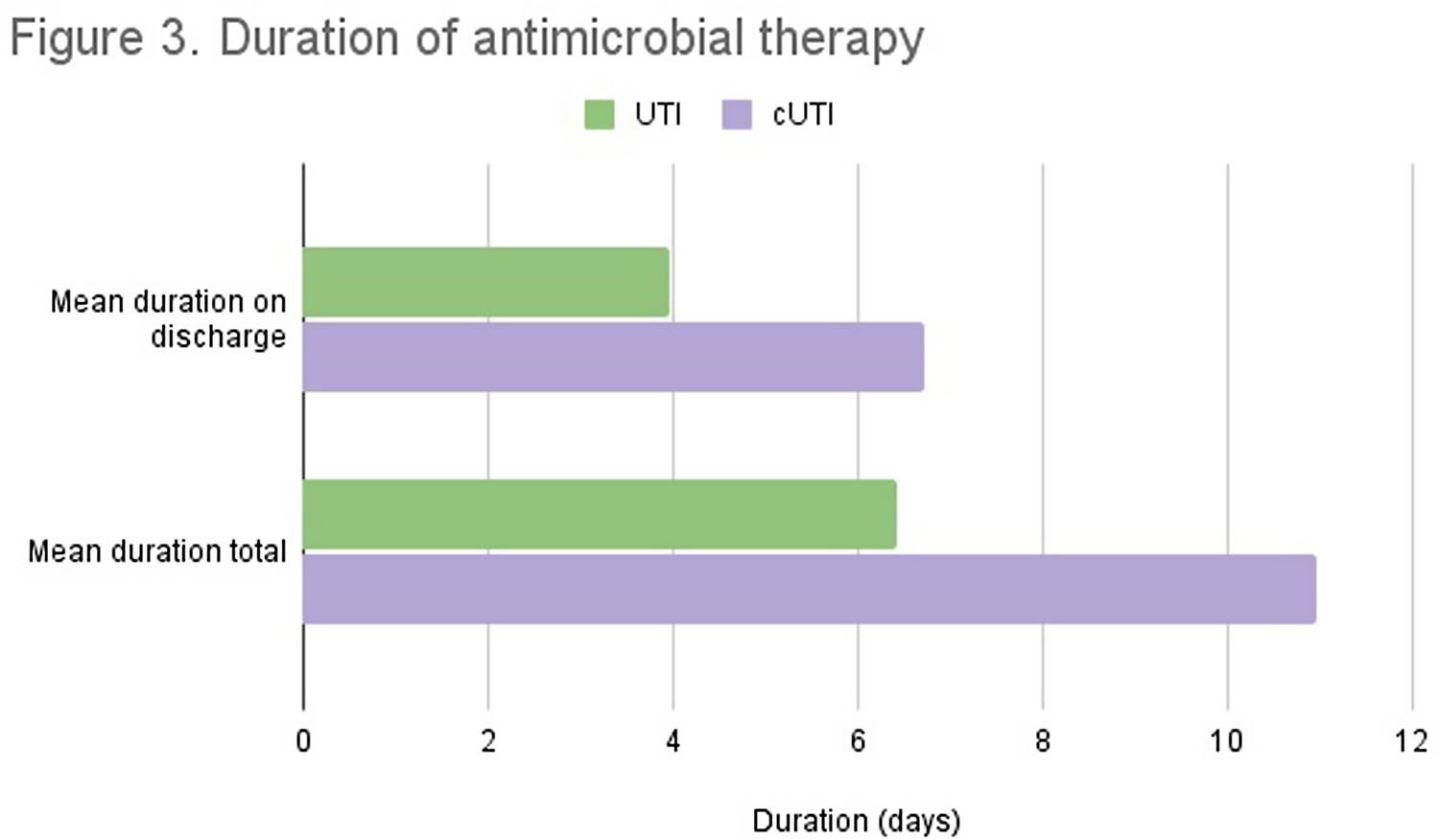# Antibiotic Prescribing Practices on Hospital Discharge for Management of Urinary Tract Infections: A Single Center Study

**DOI:** 10.1017/ash.2024.146

**Published:** 2024-09-16

**Authors:** Thomas Erwes, Melissa Colaluca, Ajshe Zulfi, Dora Wiskirchen, Jessica Abrantes-Figueiredo

**Affiliations:** UConn Health Department of Infectious Diseases; The University of Connecticut; University of Connecticut School of Medicine; Saint Francis Hospital and Medical Center

## Abstract

**Background:** Appropriate antibiotic use has been described as one of the key strategies in tackling antibiotic resistance. Although the majority of antimicrobial therapy is completed following discharge, there lacks clear guidance in addressing antibiotic stewardship in the outpatient setting. Particularly, broader coverage as well as longer durations of therapy are often encountered following hospitalization. In our study we examine the various antibiotic prescribing practices on hospital discharge for management of urinary tract infections (UTI). **Methods:** We conducted a single-center, retrospective observational chart review of patients discharged from St. Francis Hospital and Medical Center in Hartford between May and July 2022. Medical records were reviewed for patients who were prescribed antibiotic therapy for management of UTI and met inclusion criteria. Variables of interest included type of UTI treated, antibiotic used, duration of antibiotics during and following hospitalization, fluoroquinolone use, as well reported adverse events. Total duration of therapy was defined as days on susceptible antimicrobials with appropriate source control. **Results:** A total of 84 patients met inclusion criteria. 44 received treatment for simple UTI (sUTI) and 40 for complicated UTI (cUTI). Figure 1 shows the various organisms identified on culture. The most common antimicrobials prescribed on discharge were cefpodoxime and ciprofloxacin [figure 2]. Quinolones were prescribed in 11.4% of sUTIs and 39.1% of cUTIs on hospital discharge. Of those, only one patient had no alternative to quinolone use due to drug allergies. The mean duration of therapy for treatment of sUTI was 6.4 days total (SD 2.40) with 3.9 days outpatient (SD 1.78). The mean duration of therapy for treatment of cUTI was 10.9 days total (SD 3.62) with 6.7 days outpatient (SD 2.99). Comparison of mean durations is shown in figure 3. In 49% of all cases (including both sUTI and cUTI) patients received greater than 7 days of antimicrobial therapy. **Conclusion:** There is increased evidence favoring shorter courses of antimicrobial therapy for management of both simple and complicated UTIs. A 7-day course has been shown as effective duration of therapy for cUTI with appropriate source control, regardless of presence of bacteremia. Results from our single center-study show both sUTI and cUTI are subject to unnecessarily prolonged durations of therapy on hospital discharge. In addition we noted a significant use of fluoroquinolones in cUTI treatment. We believe stewardship interventions at time of discharge may particularly benefit shorter courses of therapy for cUTI as well as reduced quinolone use.

**Figure:**
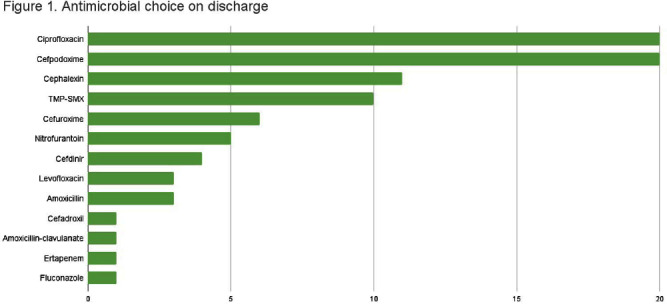

**Figure:**
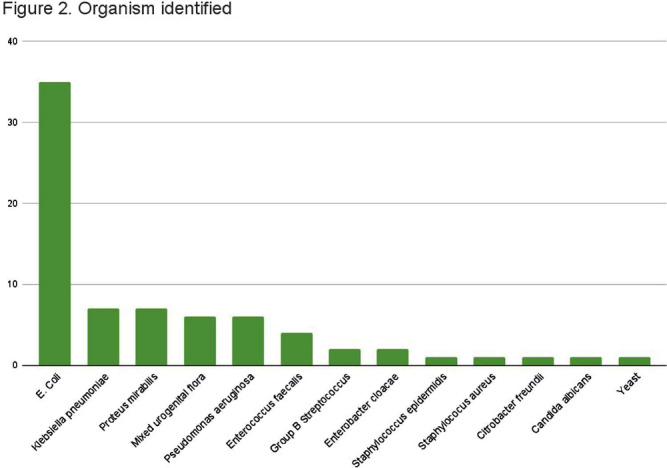

**Figure:**